# Early Detection of Fetal Malformation, a Long Distance Yet to Cover! Present Status and Potential of First Trimester Ultrasonography in Detection of Fetal Congenital Malformation in a Developing Country: Experience at a Tertiary Care Centre in India

**DOI:** 10.1155/2015/623059

**Published:** 2015-11-23

**Authors:** Namrata Kashyap, Mandakini Pradhan, Neeta Singh, Sangeeta Yadav

**Affiliations:** Department of Maternal and Reproductive Health, Sanjay Gandhi Post Graduate Institute of Medical Sciences (SGPGIMS), Lucknow 226 014, India

## Abstract

*Background*. Early detection of malformation is tremendously improved with improvement in imaging technology. Yet in a developing country like India majority of pregnant women are not privileged to get timely diagnosis.* Aims and Objectives*. To assess the present status and potential of first trimester ultrasonography in detection of fetal congenital structural malformations.* Methodology*. This was a retrospective observational study conducted at Sanjay Gandhi Postgraduate Institute of Medical Sciences. All pregnant women had anomaly scan and women with fetal structural malformations were included.* Results*. Out of 4080 pregnant women undergoing ultrasound, 312 (7.6%) had fetal structural malformation. Out of 139 patients who were diagnosed after 20 weeks, 47 (33.8%) had fetal structural anomalies which could have been diagnosed before 12 weeks and 92 (66.1%) had fetal malformations which could have been diagnosed between 12 and 20 weeks.* Conclusion*. The first trimester ultrasonography could have identified 50% of major structural defects compared to 1.6% in the present scenario. This focuses on the immense need of the hour to gear up for early diagnosis and timely intervention in the field of prenatal detection of congenital malformation.

## 1. Introduction

Fetal structural malformations are seen in 3 to 5% of all pregnancies [1]. Detection of malformation is tremendously improved with improvement in imaging technology. In majority of countries worldwide, second trimester scan between 18 and 22 weeks remains the standard of care for fetal anatomical assessment; however, most recent literature shows a significant improvement in detection of fetal abnormalities in first trimester of pregnancy [2]. Besides nuchal abnormalities a wide range of central nervous system, heart, anterior abdominal wall, urinary tract, and skeletal abnormalities can be diagnosed between 11 and 14 weeks of scan. The clear benefits of first trimester ultrasound are early detection and exclusion of major congenital anomalies (not compatible with life or followed by severe handicap), reassurance, and relatively easier pregnancy termination if required.

Currently, the review of recent literature suggests classification of fetal abnormalities as always detectable, potentially detectable, and undetectable till first trimester and anomaly scan. The diagnostic efficacy of first trimester anomaly scan and echocardiography between 11 and 14 weeks has been assessed in medium risk population by Becker and Wegner [3]. The prevalence of major anomalies in their study group was 2.8%. The overall detection rate of fetal anomalies including cardiac defects was 84% and increased with raised nuchal thickness particularly more than 2.5 mm. This highlights the scope of first trimester scan apart from its conventional role in detection of chromosomal abnormality.

First trimester screening is now no more limited to detection of raised nuchal thickness (NT). Becker [4] et al. analysed 6879 cases to assess the prevalence and detection rate of major anomalies by applying first trimester anomaly scan and fetal echocardiography. They concluded that a significant number of fetal anomalies occur with normal NT and more than half of them could be detected in first trimester. Hence, even fetuses with normal NT should be offered first trimester anomaly scan and fetal echocardiography considering the ethical principles of nonmaleficence, justice, and respect for autonomy of pregnant women. Even in this era the benefits of this established technology are not in the reach of all. A vast majority of patients in India are not yet undergoing anomaly scan. We frequently encounter malformations always or potentially detectable during first trimester scan at third trimester or in postnatal period. It depends on both the expertise and resources available along with the awareness and sensitization in general population. This fact of diagnosis is particularly more important in countries like India where medical termination of pregnancy [5] is legally allowed up to 20 weeks of gestation irrespective of malformation being lethal. We see a fair number of patients who are diagnosed with fetal malformation beyond 20 weeks and in that situation they are forced to seek termination services at small substandard centres since they get refusals from all relatively good hospitals due to legal issues associated with termination. Many of such patients get deteriorated due to septic abortion and unnecessary hysterotomy and so forth. Question then arises that where lies the fault, the awareness of the patients or the expertise of the sonologist.

Henceforth, the study was planned to assess the prevalence of fetal malformation in a tertiary care referral centre and to assess the present status of first trimester ultrasonography in the detection of fetal malformations in a tertiary care centre in India.

## 2. Materials and Methods

This was a retrospective observational study conducted at Sanjay Gandhi Postgraduate Institute of Medical Sciences. All pregnant women attending Department of Maternal and Reproductive Health, OPD, from August 2009 till October 2013 were enrolled in the study. All pregnant women underwent ultrasound (General electrical Voluson S8) and those with fetal structural malformations were evaluated. Malformations were classified according to gestational age of diagnosis, system involved, and type of malformation. Descriptive proportions and frequencies have been used to depict the data.

## 3. Results

A total number of 4080 pregnant women underwent USG and amongst them 312 (7.6%) patients had fetal structural malformation. The malformations were classified according to gestational age as shown in Table 1. Malformations were classified according to various systems as shown in Table 2.

### 3.1. Malformations Detected prior to 20 Weeks

Out of total malformed fetuses, 103 (33%) were detected prior to 20 weeks of gestational age and 209 (66.9%) were detected after 20 weeks of gestational age. Out of 103 women who were diagnosed with fetal malformations before 20 weeks, only 5 (1.6%) were detected prior to 12 weeks of gestational age and the remaining 98 (31.4%) were diagnosed between 12 and 20 weeks. Six patients amongst them presented before 12 weeks but malformations were missed and diagnosed later between 12 and 20 weeks. These cases were omphalocele, osteogenesis imperfecta, harlequin ichthyosis, Stickler syndrome, Fraser syndrome, and Dandy-Walker malformation. These conditions, however, are known to present late.

**Figure 1:**
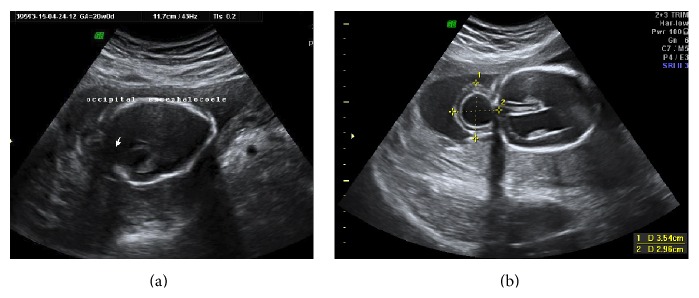
Occipital encephalocoele diagnosed at 20 weeks which was terminated.

Out of 103 patients diagnosed to have malformation prior to 20 weeks, 80 patients willingly underwent termination of pregnancy in view of malformation being lethal like a fetus with occipital encephalocoele terminated at 20 weeks of gestational age (Figure 1). We had prescribed protocol of oral mifepristone (200 mg) followed by misoprostol induction after 48 hours of mifepristone. Three patients were lost to follow-up. Ten patients had nonlethal malformation and were willing to continue pregnancy. All of them had postpartum neonatal intervention in the Department of Pediatric Surgery, Neonatology and Plastic Surgery, respectively (for posterior urethral valve, extra lobar sequestration, tracheoesophageal fistula, anorectal malformation, congenital diaphragmatic hernia with good LH ratio, meningocele, polycystic kidneys, megacystis, vesicoureteral reflux, and cleft lip palate).

Ten patients refused to continue pregnancy despite malformation being lethal. They had obstetrical procedure at their convenient places. Four amongst them had preterm still birth and six babies died in neonatal period. Biggest agony is that two amongst those continuing pregnancies with known lethal malformations had hysterotomy and two had cesarean section for anomalous fetus which could have been avoided.

We found that with the present available technology majority of malformation could be diagnosed before 20 weeks (Box 1).

First trimester sonography has huge potential of diagnosing fetal anomalies. We found that there are few malformations which could be easily diagnosed before 12 weeks (Box 2).

Five patients were diagnosed prior to 12 weeks for neural tube defect, holoprosencephaly, gastroschisis, cystic hygroma, and anencephaly. All of them had easy termination of pregnancy.

### 3.2. Malformations Detected after 20 Weeks

Out of 312 pregnant women with malformations, 209 (66.9%) were diagnosed after 20 weeks. 109 had their first USG after 20 weeks and 100 had USG prior to 20 weeks but malformations were missed.

Out of those 100 patients, 6 patients presented to our institute before 20 weeks and malformations were not confirmed until 24 weeks. In 94 women, they went for USG prior to 20 weeks at some other centre and malformation was missed. Amongst those six patients who presented to SGPGI prior to 20 weeks but were missed, there were one case each of Dandy-Walker malformation, autosomal dominant polycystic kidneys, late onset hydrocephalus, and tetralogy of Fallot. All of these tend to be diagnosed late. Two fetuses, one with cleft lip and one with neural tube defect, could have been diagnosed but were missed. There exists a group of malformation which lies in the grey zone of diagnosis before 20 weeks (Box 3).

**Figure 2:**
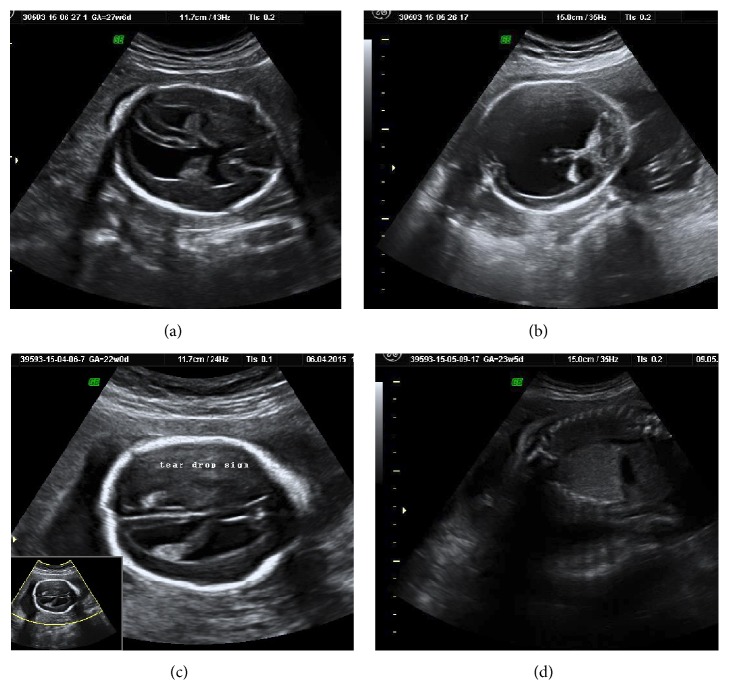
(a and b) show late onset hydrocephalus diagnosed at 27 weeks and 32 weeks, noted to occur late as a part of MASA syndrome (patient had history of X-linked hydrocephalus). (c) shows agenesis of corpus callosum which is known to be said with confirmation at later gestation. (d) shows fetal micro cystic congenital cystic adenomatoid malformation (CCAM) which is notorious to be missed early.

Out of 209 detected cases after 20 weeks, 70 (33.4%) patients had malformations which were detected after 20 weeks and are acceptable because these include conditions which tend to be diagnosed late in gestation like hydrocephalus (Figures 2(a) and 2(b)), agenesis of corpus callosum (Figure 2(c)), congenital cystic adenomatoid malformation (Figure 2(d)), various cardiac structural malformations, cystic kidney diseases, horseshoe kidney, Dandy-Walker malformations and variants, vein of Galen aneurysm, duodenal atresia, fetal goitre, intra-abdominal tumours, gonadal cyst, Hirschsprung disease, and isolated fetal ascites.

### 3.3. Malformation Missed

**Figure 3:**
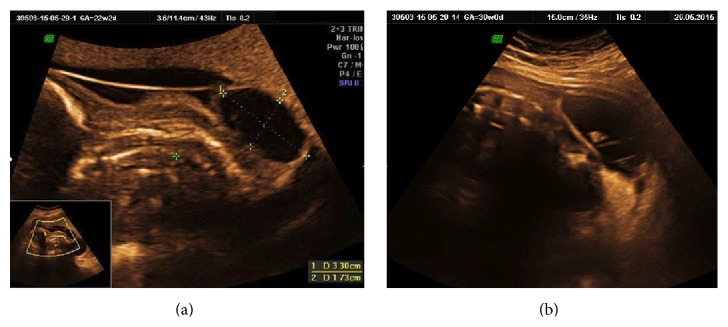
(a and b) show two cases of neural tube defect diagnosed at advanced gestational ages of 22 weeks and 39 weeks.

**Figure 4:**
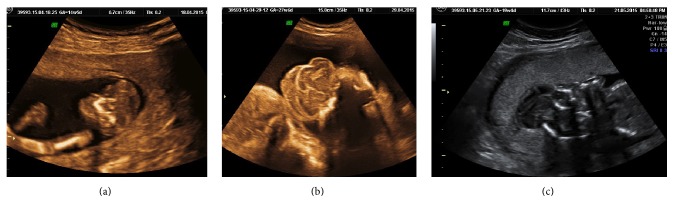
(a, b, and c) show fetal exencephaly that could be diagnosed at 14 weeks but was missed and diagnosed at 19 weeks and as late as 27 weeks.

**Figure 5:**
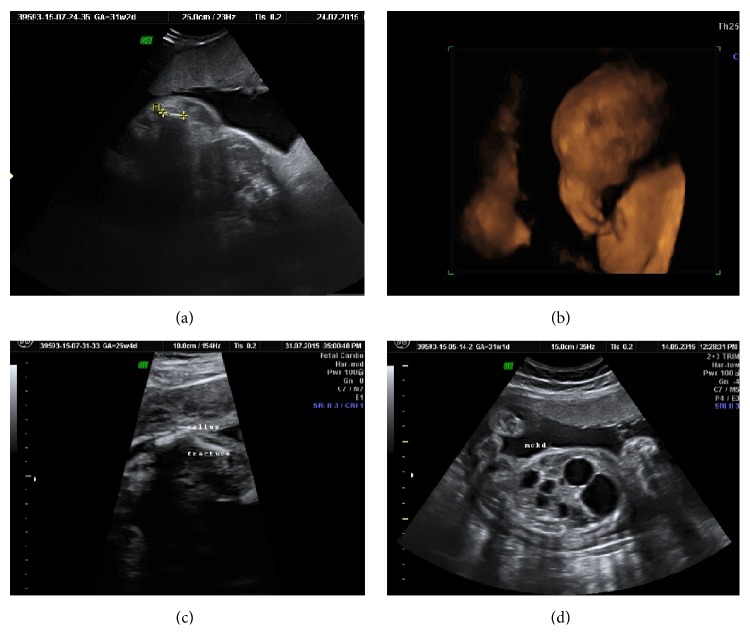
(a, b, and c) show skeletal malformation achondrogenesis (3D), thanatophoric dysplasia, and osteogenesis imperfecta. All of them potentially diagnosable before 12 weeks and usually before 20 weeks were missed and were diagnosed late. (d) shows bilateral multicystic dysplastic kidney diagnosed at 31 weeks, with fetus being continued as oligohydramnios.

**Figure 6:**
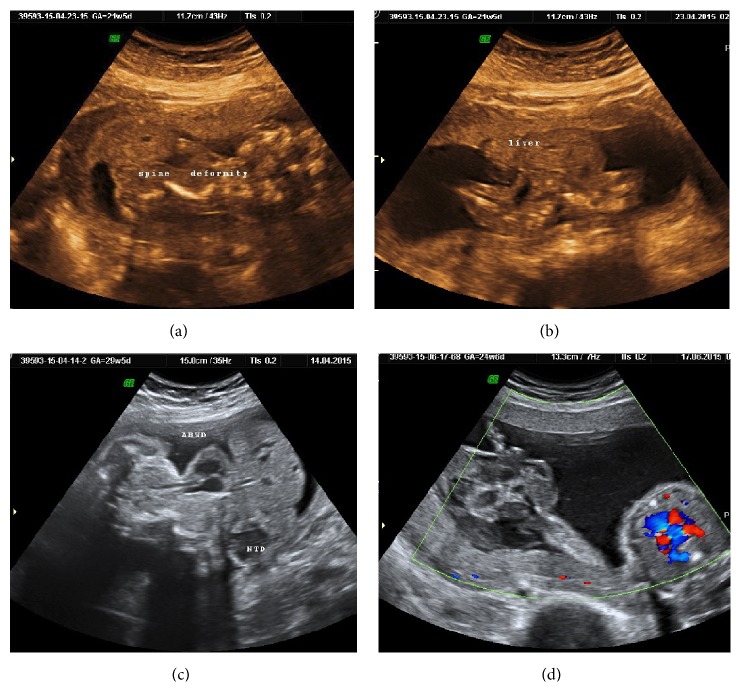
(a, b, and c) show gross fetal deformity limb body wall complex, diagnosis delayed till 21 weeks in one fetus (Figures 6(a) and 6(b)) and 29 weeks in other fetuses (Figure 6(c)). (d) shows acardiac twin (twin reverse arterial perfusion sequence) diagnosed at 25 weeks when the normal twin had already decompensated making any intervention difficult.

Even though missed in first trimester, in 139 (66.5%) patients, fetal malformations could have been diagnosed between 12 and 20 weeks as shown in Box 1. These included malformations like neural tube defect (Figures 3(a) and 3(b)), acrania-exencephaly-anencephaly sequence (Figures 4(a), 4(b), and 4(c)), skeletal dysplasia (Figures 5(a), 5(b), and 5(c)), multicystic dysplastic kidneys (Figure 5(d)), and limb body wall complex (Figures 6(a), 6(b), and 6(c)).

Prenatal interventions in very unique complications of monochorionic twins have become the treatment of choice [6] but diagnosis of acardiac twinning was delayed till 24 weeks (Figure 6(d)). This delayed pick-up of these potentially salvageable conditions leads to high likelihood of adverse pregnancy outcome. The patient had demise of normal cotwin also at 28 weeks.

## 4. Discussion

The overall prevalence of severe and lethal fetal structural malformation in our study was 7.6% which was higher than that reported in the literature for general population (3–5%), possibly because it was a referral centre for high risk pregnancy and fetal medicine; there is overreporting of cases. Our study calculated that CNS malformations were most common in our study population. As such, preconceptional folic acid is not commonly practiced in our study population. We realize that almost half (52.1%) of our patients had their first USG for anomaly detection after 20 weeks. It reflects the existing darkness of unawareness and vacuum of knowledge in patients and also in basic health care that are first to encounter pregnant women.

We found that, out of the total number of women with diagnosed fetal malformation, 203 (65%) presented before 20 weeks. Hence, equally important is the fact to realize that almost half of these patients who had malformations detected after 20 weeks had their obstetrical sonography before 20 weeks and were missed. This missing out of an anomaly may be because of scarcity of good resolution machines, busy schedules, and lack of expertise as well. For years together, there have been substantial advances in magnification imaging and signal processing which increased the ability to visualize fetal anatomy; there has been great concern on the possibility to diagnose a wide range of fetal anomalies at the time of nuchal translucency scan by transvaginal and transabdominal sonography [7–9].

Almost half of malformations in our study were amenable to be diagnosed in first trimester as reported in current literature. These fetuses were having malformations like neural tube defects, anencephaly, holoprosencephaly, and gastroschisis (Box 2). Castro-Aragon and Levine [10] reported that 60–67% of malformations could have been diagnosed prior to 12 weeks. This is far away from our scenario where we found that only 1.6% (5/312) were diagnosed prior to 12 weeks. This is possibly due to the lack of awareness and lack of expertise as well. Fong et al. [11] in their study scanned 8,537 women between 11 and 14 weeks of gestation (crown rump length, 45–84 mm); there were 175 fetuses with an increased NT. Besides nuchal abnormalities, a wide range of other congenital anomalies can be diagnosed with US at 11–14 weeks of gestation, including defects of the central nervous system, heart, anterior abdominal wall, urinary tract, and skeleton. Oztekin et al. [12] analyzed 1085 pregnancies; 21 (1.93%) fetuses had at least one major structural defect considered detectable by routine ultrasound screening. 14 (1.29%) were identified at early (first trimester) screening and an additional 5 (0.47%) were identified at late (second trimester) USG. They found that majority of fetal structural abnormalities can be detected by sonographic screening at 11–14 weeks, but detailed fetal anatomic survey performed at 18–22 weeks should not be abandoned.

Rossi and Prefumo [13] also laid stress that first trimester ultrasound can detect half of fetal malformations. They included nineteen studies on 78,002 fetuses, with 996 with malformations that were confirmed by postnatal or postmortem examinations. USG at 11 to 14 weeks detected malformation in 472 of the malformed fetuses (51%). Detection rate was highest for neck anomalies (92%) and lowest for limbs, face, and genitourinary anomalies (34% for each). The presence of associated anomalies appears to increase the accuracy of early ultrasonography. Multiple defects were more likely to be identified than isolated malformations (60% versus 44%). Detection rates ranged from 1% to 49% for spina bifida or hydrocephalus, ranged from 50% to 99% for valvular disease and septal defects, were 100% for acrania and anencephaly, and were 0% for corpus callosum agenesis and bladder exstrophy. Combination of transabdominal and transvaginal techniques resulted in a 62% detection rate versus 51% for transabdominal technique only and 34% for transvaginal technique only.

Although first trimester ultrasound can detect about 50% of fetal malformations, it cannot replace second trimester ultrasound because several malformations develop later than the first trimester. Also to be kept in mind is the fact that accuracy of early ultrasonography can be compromised by transient findings like midgut herniation, small septal defects, and hydronephrosis which might get resolved during intrauterine life.

Iliescu et al. [14] did a prospective two-centre 2-year study of 5472 consecutive unselected pregnant women examined at 12 to 13 + 6 gestational weeks. The first trimester scan identified 40.6% of the cases detected overall and 76.3% of major structural defects. Major congenital heart disease (either isolated or associated with extracardiac abnormalities) was 90%. Major central nervous system anomalies were 69.5%. Fetuses with increased nuchal translucency (NT), the first trimester DR for major anomalies, were 96% compared to 66.7% amongst those with normal NT.

There have been several studies seeing application for an extended protocol in which first trimester sonography is supported by a second anomaly scan. The obvious advantage of an extended protocol is that parents are offered the option of earlier and safer termination of pregnancy for the large majority of severe/lethal abnormalities.

Early ultrasound might be more accurate than second trimester ultrasonography for detection of malformations associated with oligohydramnios and anhydramnios which lead to poor visualization at later gestation necessitating amnioinfusion.

We have applied this kind of protocol at our centre particularly in high risk women. First trimester sonography with targeted imaging for fetal malformation appeared particularly more helpful in high risk women with previous history of fetus/neonates with malformations, known risk factors, for example, type 2 diabetes, patients prone for teratogenicity, for example, thromboembolic and valve replacement patients on warfarin, methotrexate intake for connective tissue disorders, and so forth, antiepileptic, chemotherapeutic drugs, and history of infection exposure like rubella. We diagnosed and terminated patients before 16 weeks with rubella exposure and subsequent pulmonary stenosis, severe bony stippling and craniofacial malformation associated with high doses warfarin, VSD associated with type 2 diabetes, neural tube defect with antiepileptic and tetraphocomelia with chemotherapeutic agents, renal agenesis in previous history of Fraser's syndrome, encephalocoele in previous Meckel-Gruber syndrome, ARPKD and ADPKD, and so forth.

However, a detailed first trimester examination protocol involves supplementary resources: additional examination time and specialized personnel for the abnormal suspected/detected cases. Healthcare systems are yet to determine whether early first trimester diagnosis of most major structural abnormalities is cost-effective. Previous research, albeit using inferior ultrasound technology and a less extended protocol, found that the first trimester anomaly scan was cost-efficient in terms of medical and economic expenses, although they obtained lower detection rates [15, 16].

The present research about the effectiveness of early ultrasonography in the diagnosis of structural defects does have some conflicts, which made it a challenge that to what extent structural congenital abnormalities could be detected by the routine scanning of fetal anatomy combined with nuchal translucency measurement [17]. Few other basic prerequisites associated with early prenatal diagnosis consist of the high experience required and high costs in terms of time and equipment [18]. Even with all these circumstances, the situation in our country is such that a huge number of patients, 209 (66.9%), were diagnosed after 20 weeks which shows the lacunae which need to be filled.

## 5. Conclusion

In our study we realized that even in a tertiary care centre only 1.6% fetuses with malformation are identified in first trimester. In a way, it throws light on the importance of screening as well as an immense need for early diagnosis and timely intervention in the field of prenatal detection of congenital malformation.

A detailed examination of fetal anatomy during the routine 11–14 weeks of gestation scan provides a comprehensive assessment of fetal anatomy and can detect approximately half of major structural defects in both low-risk and high-risk pregnancies. Detection rate increases markedly beyond 13 weeks of gestation compared with 11 weeks of gestation. We have seen to be better convinced to diagnose holoprosencephaly, achondrogenesis, osteogenesis imperfecta, and spondylocostal dysostosis at 14 weeks compared to 12 weeks. It is also expected that because of the late development of some organ systems and the delayed onset of a significant number of major anomalies in the second and third trimester it is very unlikely that the early scan may replace second trimester ultrasonography.

We need to identify structural malformations before 20 weeks except those conditions which are said to appear further late or reported with confirmation at a later gestational age like few posterior fossa abnormalities, duodenal atresia, and few renal abnormalities. The most important implication is safe termination and avoiding maternal threat to life by forced termination at resourceless and substandard centres. There could be an option of incorporating anomaly scan between 18 and 20 weeks in our health plans and guides at well registered centres with expertise at reasonable cost.

Focus and emphasis should aim at detection of malformation earlier than 12 weeks owing to the very unique and clear facts that first trimester detection leads to easy termination of pregnancy and lessening of women's mental, physical, and psychological trauma.

## Figures and Tables

**Box 1:**
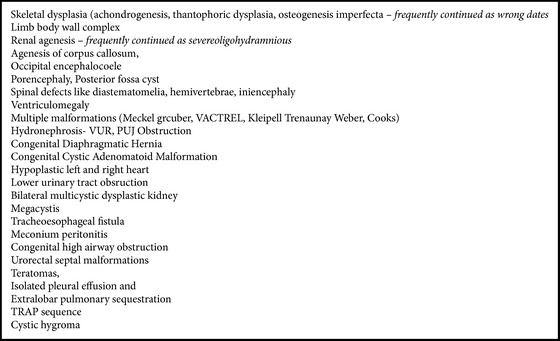
List of common malformations detected before 20 weeks.

**Box 2:**
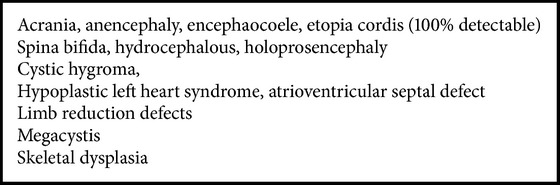
List of common malformations we found usually detectable before 12 weeks.

**Box 3:**
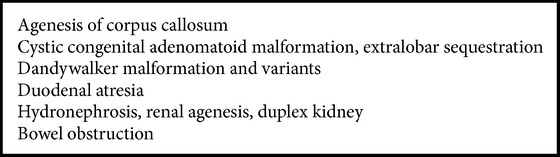
List of malformations we found undetectable before 12 weeks and difficult between 12–20 weeks.

**Table 1:** Number of malformations at different gestational age.

Gestational age	Number
<12 weeks	5
12–20 weeks	98
>20 weeks	209

**Table 2:** Classification of malformation according to the system involved.

CNS, brain	Number	Skeletal	Number
Neural tube defects	30	Achondroplasia	2
Porencephaly	1	Hypochondroplasia	2
Anencephaly	11	Osteogenesis imperfecta	3
Occipital encephalocoele	8	Short rib polydactyly	6
Iniencephaly	2	Thanatophoric dysplasia	1
Ventriculomegaly	14	Single forearm bone	1
Arachnoid cyst	2	Cooks syndrome	1
Holoprosencephaly	8	*Respiratory *	
Agenesis of corpus callosum	8	CCAM	7
Dandy-Walker malformation	16	Pleural effusion	1
Diastematomyelia	2	Congenital high airway obstruction	3
Vermian agenesis	2	Extralobar pulmonary sequestration	2
*Genitourinary *		*Heart*	
ADPKD	3	Structural cardiac malformations	44
ARPKD	1	Congenital heart blocks	8
Megacystis	1	Pericardial effusion	2
Gonadal cyst	2	Structural and rhythmic	1
Hydronephrosis	12	*AV malformation*	
Lower urinary tract obstruction	9	Vein of Galen malformation	1
Horseshoe kidney	1	Klippel-Trenaunay-Weber syndrome	1
Unilateral multicystic kidney	17	*Others*	
Bilateral cystic kidney disease	9	Fetal goitre	1
*Gastrointestinal *		Cystic hygroma	6
Fetal ascites	1	*Multiple*	
Meconium peritonitis	2	Limb body wall complex	2
Gastroschisis	3	VACTERL	1
Mesenteric cyst	1	Meckel-Gruber syndrome	6
Enteric duplication cyst	1	Multiple malformations	20
Hirschsprung disease	1	*Fetal tumors*	
Tracheoesophageal fistula	2	Adrenal neuroblastoma	1
Congenital diaphragmatic hernia	3	Teratoma	3
Duodenal atresia	4	Sacrococcygeal teratoma	1
Isolated fetal ascites	2		
Omphalocele	6		
